# Myocardial Perfusion Scintigraphy Provides Incremental Prognostic Value in Patients on the Kidney Transplant Waiting List

**DOI:** 10.1111/ctr.70114

**Published:** 2025-02-21

**Authors:** Stefan Reuter, Stefanie Reiermann, Jörg Stypmann, Joachim Bautz, Katharina Schütte‐Nütgen, Hermann Pavenstädt, Viola Malyar, Holger Reinecke, Marc‐Andre Kurosinski, Dennis Görlich, Hans‐Werner Hense, Barbara Suwelack, Michael Schäfers

**Affiliations:** ^1^ Department of Internal Medicine D University Hospital Münster Münster Germany; ^2^ Department of Cardiology I – Coronary and Peripheral Vascular Disease Heart Failure University Hospital Münster Münster Germany; ^3^ Department of Nuclear Medicine University Hospital Münster Münster Germany; ^4^ Cells in Motion Interfaculty Centre University of Münster Münster Germany; ^5^ Institute of Epidemiology and Social Medicine University of Münster Münster Germany; ^6^ Institute of Biostatistics and Clinical Research University of Münster Münster Germany

**Keywords:** diagnostic techniques and imaging, echocardiography, kidney transplantation, living donor, waitlist management

## Abstract

The approach to cardiovascular risk assessment before renal transplantation is still controversial. Therefore, we evaluated and compared the prognostic value of myocardial perfusion scintigraphy (MPS) and dobutamine stress echocardiography (DSE) in patients with end‐stage renal disease (ESRD) who are candidates for kidney transplantation (KTx). Methods: We prospectively enrolled 356 ESRD clinical transplantations for review, only patients (NCT01064674) admitted to our transplant center between August 2009 and July 2012. Cardiovascular risk assessment at the time of listing was based on the Münster Cardiovascular Risk Stratification Score (MCRSS), additionally including evaluation by DSE and MPS in all ESRD patients. Coronary angiography was conducted in patients at high risk according to the MCRSS and in those where noninvasive stress testing revealed stress‐induced ischemia or wall motion abnormalities. Results: During long‐term follow‐up until October 2020, 2.43 cardiovascular events/100 person‐years (nonfatal stroke, nonfatal myocardial infarction, and cardiovascular death) occurred, and the overall patient survival was 71.9%. Mild perfusion deficits identified by MPS, unlike wall motion abnormalities detected by DSE, showed incremental prognostic value for event‐free survival in patients with low MCRSS risk. Conclusion: We therefore propose a modified MCRSS‐based approach including MPS as a reasonable risk stratification approach for cardiovascular risk assessment of ESRD patients applying for KTx.

## 1 Introduction

Chronic kidney disease significantly promotes the development of vascular calcification, atherosclerosis, and coronary artery disease (CAD), especially in patients with end‐stage renal disease (ESRD) [1, 2]. As a result, ESRD patients are at risk for myocardial infarction, heart failure (HF), and cardiac death; cardiovascular disease (CVD) remains the leading cause of death after kidney transplantation (KTx) [3, 4]. To reduce perioperative and postoperative morbidity and mortality, patients who apply for KTx require a thorough CV risk assessment [5]. Most centers perform risk stratification and further work‐up depending on the patient's individual CV risk burden. An analysis of the US Renal Data System registry data on cardiac evaluation services from 27 786 Medicare beneficiaries transplanted between 1991 and 2004 showed that 46% of patients underwent noninvasive stress testing or angiography before transplantation (only 65% of high‐risk patients, defined as diabetes mellitus, ischemic heart disease, or ≥2 other risk factors for CAD, and only 20% of patients with lower risk) [6]. In this context, we have recently shown that the Münster Cardiovascular Risk Stratification Score (MCRSS) performs reasonably to classify patients according to CV risk [7].

Contrary to many “non‐renal” CV patients, CVD has a different vascular pathology with different clinical presentation (often asymptomatic patients or with atypical ischemia symptoms) in renal patients [2, 3, 8]. In addition to the often asymptomatic CAD of large coronary arteries, microvascular disease is common in this group [3]. Therefore, identification of patients with occlusive CAD by coronary angiography may not necessarily identify patients at high risk for a CVD event after renal transplantation. However, identification and treatment of significant CAD in transplant candidates has been shown to improve patient survival [2, 9]. It should be noted that the recent ISCHEMIA‐CKD study in patients with stable CAD and advanced chronic kidney disease with moderate or severe ischemia showed no superiority of an initial invasive strategy (angiography and revascularization) over an initial conservative strategy in terms of reducing mortality or myocardial infarction [10].

Due to its different nature, it is still debatable how to assess CV risk in KTx candidates and identify patients who may benefit from further testing or coronary intervention before KTx [11, 12]. Data for noninvasive cardiac imaging, that is, myocardial perfusion scintigraphy (MPS) and dobutamine stress echocardiography (DSE) in transplant candidates are controversial [13]. Even mildly abnormal MPS results are prognostic in patients with ESRD, in contrast to populations with known CAD [13, 14].

Therefore, we herein aimed to further evaluate the potential of MPS and DSE for improved risk assessment in ESRD patients and conducted a prospective longitudinal observational study at our center between August 2009 and July 2012 including 356 patients who applied for KTx. CV risk evaluation was performed according to recommendations of the MCRSS risk algorithm, but all ESRD patients with low and intermediate MCRSS risks were also systematically tested by DSE and MPS [15, 16]. The primary endpoint was overall survival. The secondary endpoint was freedom from major cardiovascular events (myocardial infarction, stroke, or death from cardiovascular causes).

## 2 Methods

### 2.1 Subjects

We conducted a prospective longitudinal observational study including 356 adult patients who applied for KTx at our transplant center from August 2009 to July 2012 (NCT01064674). Follow‐up of each patient was censored on October 1, 2020. Exclusion criteria included patients with a history of heart transplantation before listing, with psychiatric disorders, and pregnant or breast‐feeding women. Data was extracted from medical records. Data of all patients were anonymized before analysis. All participants gave written consent at the time of listing. The study was approved by the local ethics committee (Ethik Kommission der Ärztekammer Westfalen‐Lippe und der Medizinischen Fakultät der Westfälischen Wilhelms‐Universität, No. 2009‐461‐f‐S). The methods in this study were performed in accordance with current transplantation guidelines and the Declaration of Istanbul and Helsinki.

### 2.2 CV Risk Assessment

In addition to clinical assessment and evaluation of CVD (family) history, echocardiography and electrocardiogram (ECG) were performed in each patient. Full details about MCRSS have been published previously [15, 17]. Briefly, the MCRSS classifies patients into three different CV risk categories: low, intermediate, or high risk. It depends on information about age (<50 years of age or ≥50 years of age), presence of diabetes, history of CAD/cardiac intervention or HF, ECG, physical fitness, and cardiac symptoms during exercise. Example classifications are shown in Table S1. All patients were routinely evaluated with DSE and MPS.

DSE was performed according to societies’ protocols using either the Philips IE 33 (Philips Healthcare, Hamburg, Germany) or GE VIVID 7 (GE Healthcare, Solingen, Germany) echocardiography system [18, 19]. All examinations were performed and interpreted by two experienced echocardiographers (DEGUM level III). Dobutamine was infused intravenously, starting at 10 µg/kg/min and gradually increased to 20, 30, and 40 µg/kg/min every 2 min, respectively. Quality criteria included achieving target heart rates; atropine was used when necessary. Stress‐induced wall motion abnormalities were considered as indicators of reversible ischemia.

MPS was performed according to societies’ protocols using a hybrid two‐slice SPECT/CT device (Symbia T2 TruePoint; Siemens Medical Solutions) [20, 21]. Doses of 250 MBq (stress) and 750 MBq (rest) ^99m^Tc‐tetrofosmin (Myoview; GE Company, Fairfield, CT) were applied. Myocardial stress was induced by treadmill exercise or intravenously administering adenosine 0.14 mg/kg/min for 4 min (Adenoscan, Astellas Pharma, Deerfield, IL). Gated SPECT/CT images with eight gates were acquired 30–60 min after peak‐stress/rest injection. Images were analyzed semiautomatically by Corridor4DM software, version 6.1 (INVIA, Ann Arbor, University of Michigan Medical Center), visually controlled, manually adjusted, and compared to an in‐house control group. A 17‐segment model of the American Heart Association was used for attenuation‐corrected images to detect perfusion defects. All segments were scored from zero (no perfusion defect) to four (absence of tracer uptake). Attenuation‐corrected SPECT images were evaluated by two experienced nuclear medicine physicians. Summed stress score (SSS) was used to quantify vasodilator stress‐induced perfusion and classified as normal (SSS 0%), mildly abnormal (SSS >0%–10%), or moderate‐severely abnormal (SSS >10%).

Pathological DSE or MPS findings were followed by coronary angiography. In high‐risk patients (symptomatic CAD, HF, history of myocardial infarction, coronary artery bypass grafting more than 5 years ago or percutaneous coronary intervention more than 2 years ago, or cardiac symptoms, that is, MCRSS high‐risk group), coronary angiography was performed in addition to DSE and MPS, irrespective of their results. Stenosis severity of each epicardial coronary artery was assessed visually and graded as follows: normal, sclerosis (<70% luminal narrowing), and stenosis (>70% luminal narrowing).

### 2.3 Statistical Analysis

Patients in the study were evaluated for their demographic and clinical characteristics at the time of listing for KTx. The day of listing was used as the starting point for morbidity and mortality follow‐up (time zero). Nonfatal myocardial infarction, nonfatal stroke, or cardiovascular death, whichever occurred first, was recorded as the study endpoint. Furthermore, patients were removed from follow‐up the day they received KTx–treated as a censoring event. Patients who did not experience a cardiovascular event and did not receive a KTx were censored at the end of the observation period unless they were lost to follow‐up due to deregistration or migration. The cumulative incidence of the study outcome was determined by proportional hazard models to investigate the effects on event‐free survival (EFS) and occurrence of KTx. We calculated total person‐years by summing the follow‐up time for all patients until the first event (EFS) or censoring and for all patients until death or censoring (overall survival). Event‐specific person‐years were calculated using overall survival. The statistical analysis system SAS 9.4 was used to execute all calculations.

## 3 Results

Table 1 lists the baseline characteristics of the study population. The median age at the listing was 52.5 years, 60.1% were men, and the median BMI was 26 kg/m^2^. Most patients were on dialysis (hemodialysis 77.8%, peritoneal dialysis 16.3%) for a median time of 19.5 months at the time of listing. CV risk factors were highly prevalent: hypertension 89.0%, dyslipidemia 66.0%, diabetes mellitus 20.0%, active smokers 21.0% (former smokers 33.7%), and obesity (BMI >30: 22.0%). Overt or history of CVD was observed as follows: peripheral arterial disease (PAD) in 7.9%, CAD in 11.2%, and cerebrovascular disease in 7.3% of patients.

**TABLE 1:** Patients’ demographic and clinical characteristics at baseline (day of listing).

Parameter	(*N* = 356)	*N* miss
Male sex	60.1%	0
Mean age at start of dialysis (years)	49.9	16^a^
Mean age at listing (years)	52.5	0
Smoking: active smokers	21.0%	0
Ex‐smokers	33.7%	0
History of cardiovascular disease		0
Coronary artery disease or coronary sclerosis	11.2%	
Peripheral artery disease	7.9%	
Cerebrovascular disease	7.3%	
Hypertension	89.0%	0
Diabetes mellitus	19.9%	0
Type 1	3.9%	
Type 2	14.0%	
New onset of diabetes after KTx	1.4%	
Hypercholesterolemia	66.0%	74
Dialysis	95.2%	0
Hemodialysis	77.8%	
Peritoneal dialysis	16.3%	
Both	1.1%	
Mean time from first dialysis to listing (months)	25.4	16^a^
Mean body mass index (BMI; kg/m^2^)	26.7	0
BMI <25	39.9%	
BMI 25–30	37.6%	
BMI >30	22.5%	
History of prior transplantations		0
No history of transplantation	85%	
1 Transplantation in history	13%	
2 Transplantations in history	1%	
3 Transplantations in history	1%	
4 Transplantations in history	<1%	
Transplantations during follow‐up	39.6%	
Cause of renal disease		0
Diabetes mellitus	11.2%	
Glomerulonephritis	24.7%	
Hypertension	12.9%	
Polycystic kidney disease	14.0%	
Interstitial nephritis	3.7%	
Reflux nephropathy	4.2%	
Focal segmental sclerosis	1.1%	
Vasculitis	3.4%	
Other	10.4%	
Unknown	14.3%	

*Note:* Hypertension was recorded in case of antihypertensive medical treatment; hypercholesterolemia if the patient received statin or total cholesterol was >6.2 mmol/L (240 mg/dL); history of CAD comprised previous myocardial infarction or proof by angiography; cerebrovascular disease comprised stroke or transitory cerebral ischemia; and peripheral artery disease comprised proof of plaque by imaging or verified intermittent claudication.

^a^ 16 patients with ESRD but not on dialysis. KTx, kidney transplantation; *N*, number; *N* miss, numbers missing.

Table 2 presents the stratification of patients into CV risk groups–low, intermediate, and high–based on MCRSS. According to MCRSS, most patients had an intermediate risk (53.0%), while the high‐risk category was less common (10.7%) than the low‐risk group (36.2%).

**TABLE 2:** Münster Cardiovascular Risk Stratification Score (MCRSS).

MCRSS risk category		*N*	*N* miss
		356	0
1–Low	36.2%	129	
2–Intermediate	53.0%	189	
3–High	10.7%	38	

*Note:* The score was calculated according to parameters given in [15, 17].

### 3.1 CV Assessment by DSE and MPS

Table 3 presents the results for the entire study follow‐up. Patients (*N* = 333) underwent MPS and/or DSE (*N* = 316). A total of 304 patients underwent DSE and MPS, 11 with DSE only, and 29 with MPS only. A total of 58 patients had imaging abnormalities, 37 of whom had stress‐induced ischemia in MPS (11.11% of 333 performed MPS), and 34 stress‐induced wall motion abnormalities in DSE (10.76% of 316 performed DSE). CAD was found in 24 out of 32 patients (who underwent coronary angiography) with stress‐induced ischemia in MPS and 26 out of 29 (who underwent coronary angiography) with significant findings in DSE. A stenosis (>70% luminal diameter) was observed in 16 and 18 patients, respectively, leading to intervention in 10 and 11 patients, respectively.

**TABLE 3:** Results of coronary angiography upon stress‐induced ischemia/wall motion abnormalities (WMAs) (upper part of the table) and in occasional high‐risk patients without prior stress test (lower part of the table).

	MPS (*n* = 333)	DSE (*n* = 316)
Stress‐induced ischemia/WMA	38	34
Coronary angiography	32	29
Sclerosis	8	8
Stenosis^a^	16	18
Intervention	10	11

Coronary angiography without prior stress test	*n* = 6
Without any finding	1
Sclerosis	5
Stenosis^a^	4
Intervention	3

^a^ Stenosis was defined as luminal narrowing >70%.

Six symptomatic patients with high CV risk according to MCRSS underwent coronary angiography without prior stress testing (6/38). CAD was diagnosed in five out of them (83.3%). Four out of six patients had a stenosis (66.6%) and three received intervention (50%).

### 3.2 Outcomes

Table 4 presents the incidence of CV events before KTx. In short, 100 patients (28.1%) died during the entire study period; 21 (5.9%) died due to cardiovascular events (0.74 fatal CV events/100 person‐years), 48 (13.5%) of non‐CVD, and in 31 (8.7%) of unknown cause. In total, 38 myocardial infarctions and 26 strokes occurred (1.69 nonfatal CV events/100 person‐years) in the follow‐up period.

**TABLE 4:** Cardiovascular events (*N* = 356 patients).

	*N* (%)
Total deaths	100 (28.1)
Cardiovascular death	21 (5.9)
Noncardiovascular death	48 (13.5)
Unknown cause	31 (8.7)
Cardiovascular events	61 (17.1)
Myocardial infarction (all)	38 (10.7)
Myocardial infarction (nonfatal)	31 (8.7)
Stroke(all)	26 (7.3)
Stroke (nonfatal)	19 (5.3)

Three patients experienced myocardial infarction and stroke during FU.

Patients in the cohort were followed until October 1, 2020 for the occurrence of KTx and their respective EFS. Overall, patients were followed for a median of 3439 days (95% CI), resulting in a cumulative follow‐up time of 2630 person‐years (based on follow‐up until the first event). During this observation period, 225 patients underwent transplantation. Figure S1A shows a graphical representation of the cumulative occurrence of KTx, taking into account competing events (such as a CV event or death that successively reduced the number of patients eligible for transplantation). The median waiting time from listing to transplantation was 639 days (IQR 268–2011 days). Figure S1B shows the time course of transplantations at 6‐month intervals over the entire study period.

**FIGURE 1:**
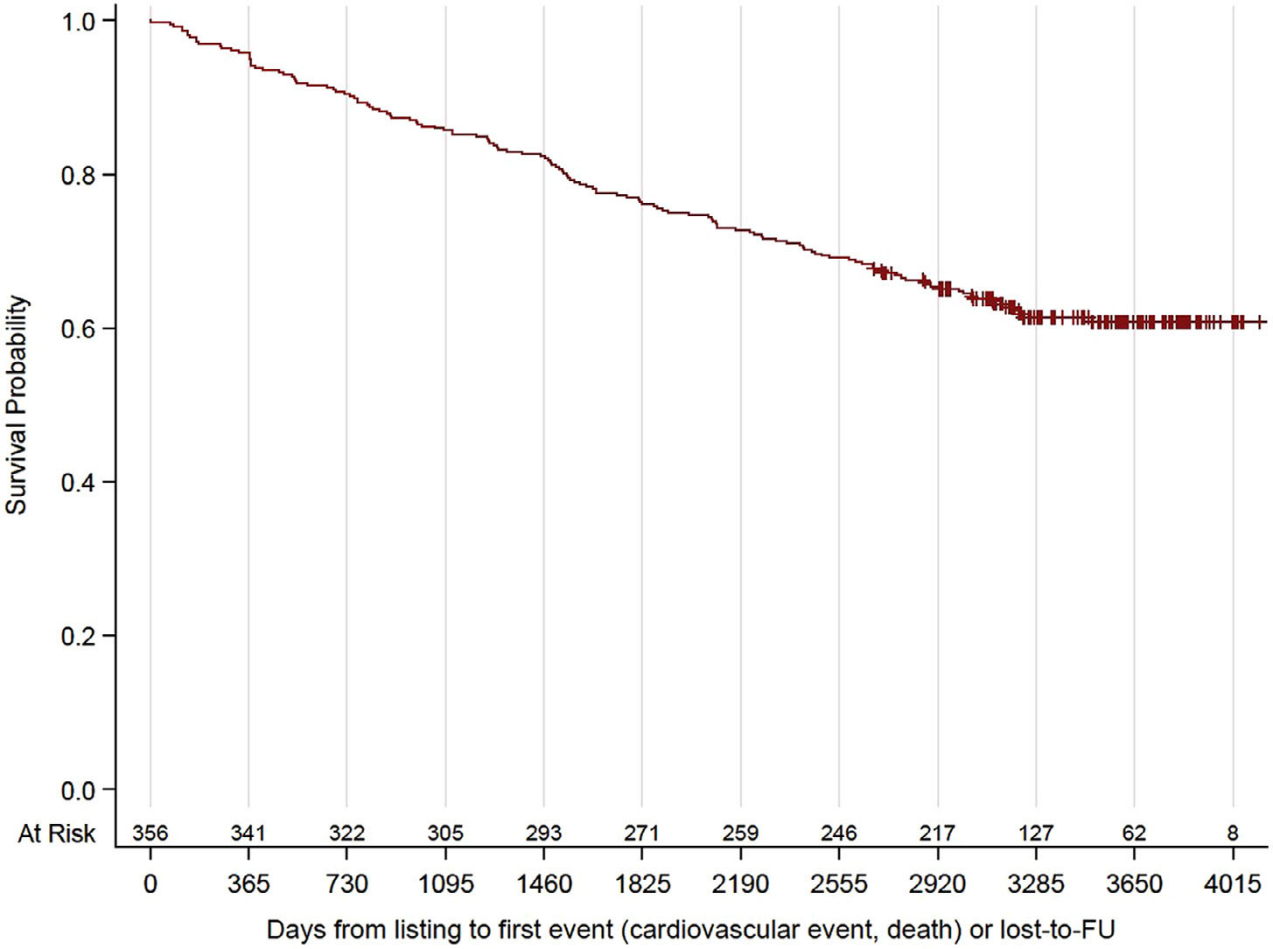
Event‐free survival of 356 patients enrolled over 4 years.

**FIGURE 2:**
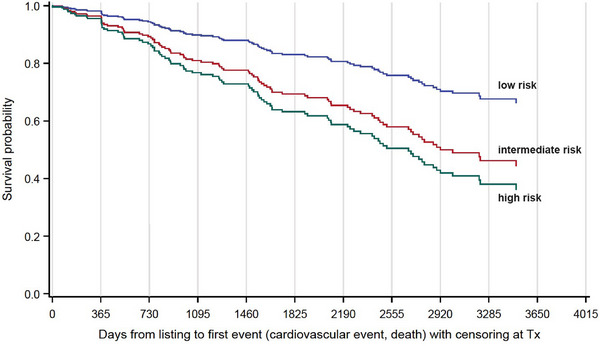
Event‐free survival of 356 patients categorized by Münster Cardiovascular Risk Stratification Score (MCRSS) into low, intermediate, and high‐risk groups.

**FIGURE 3:**
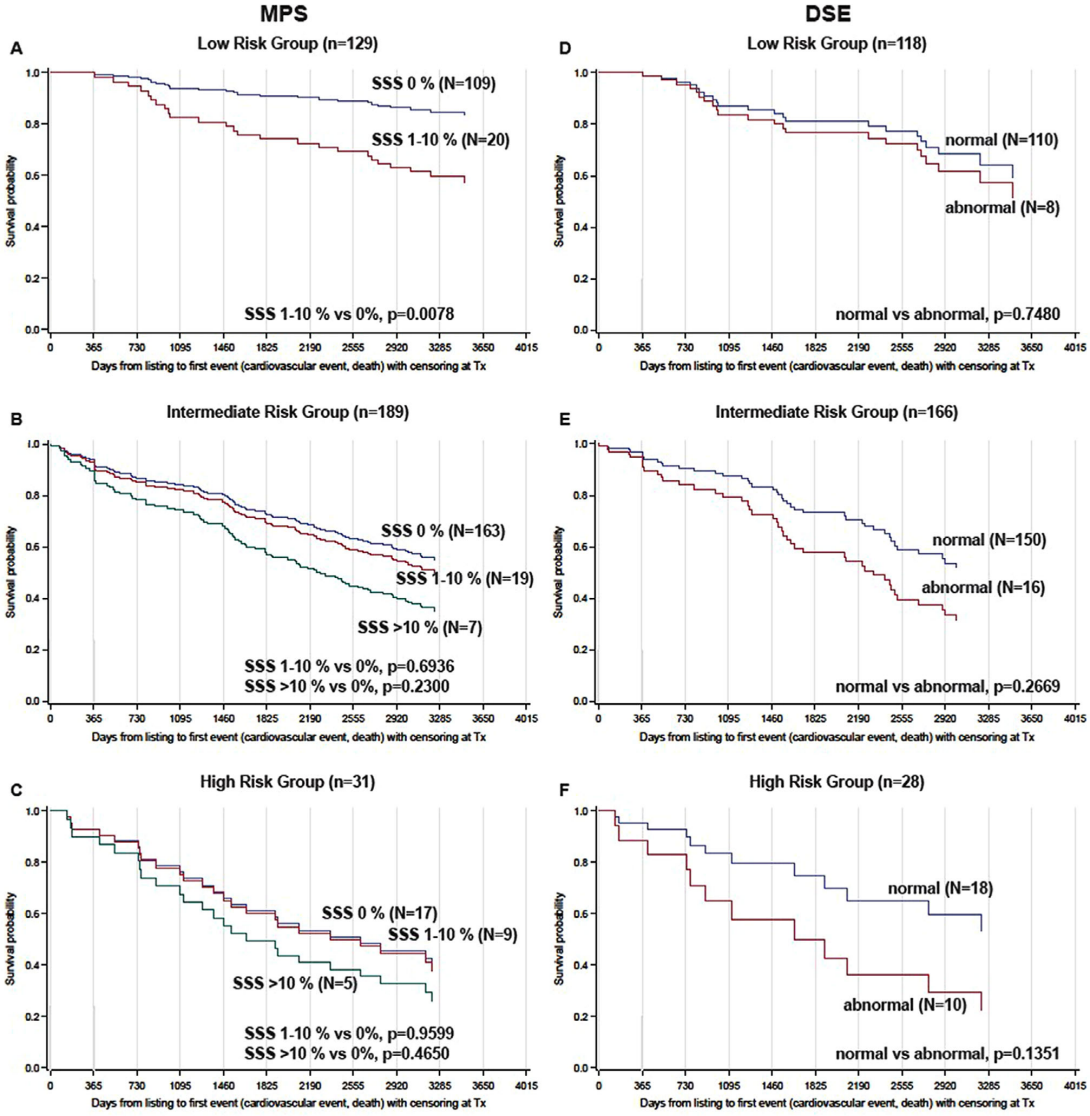
Time from listing to first event or censoring. MPS‐ (left panel) and DSE‐based (right panel) CV risk evaluation. Analyses of patients with low (A, D), intermediate (B, E), and high (C, F) MCRSS‐based CV risk. SSS assesses scarring and reversible ischemia. MPS was defined as normal if SSS was 0% (green lines) and pathologic if perfusion defects occurred. SSS was classified as mild (SSS; 1%–10%, blue lines) and moderate‐severely abnormal (SSS >10%, red lines). Patients with stress‐induced wall motion abnormalities in DSE are represented by blue lines and without abnormalities with red lines (right panel). DSE, dobutamine stress echocardiography; MCRSS, Münster Cardiovascular Risk Stratification Score; MPS, myocardial perfusion scintigraphy; SSS, summed stress score.

**FIGURE 4:**
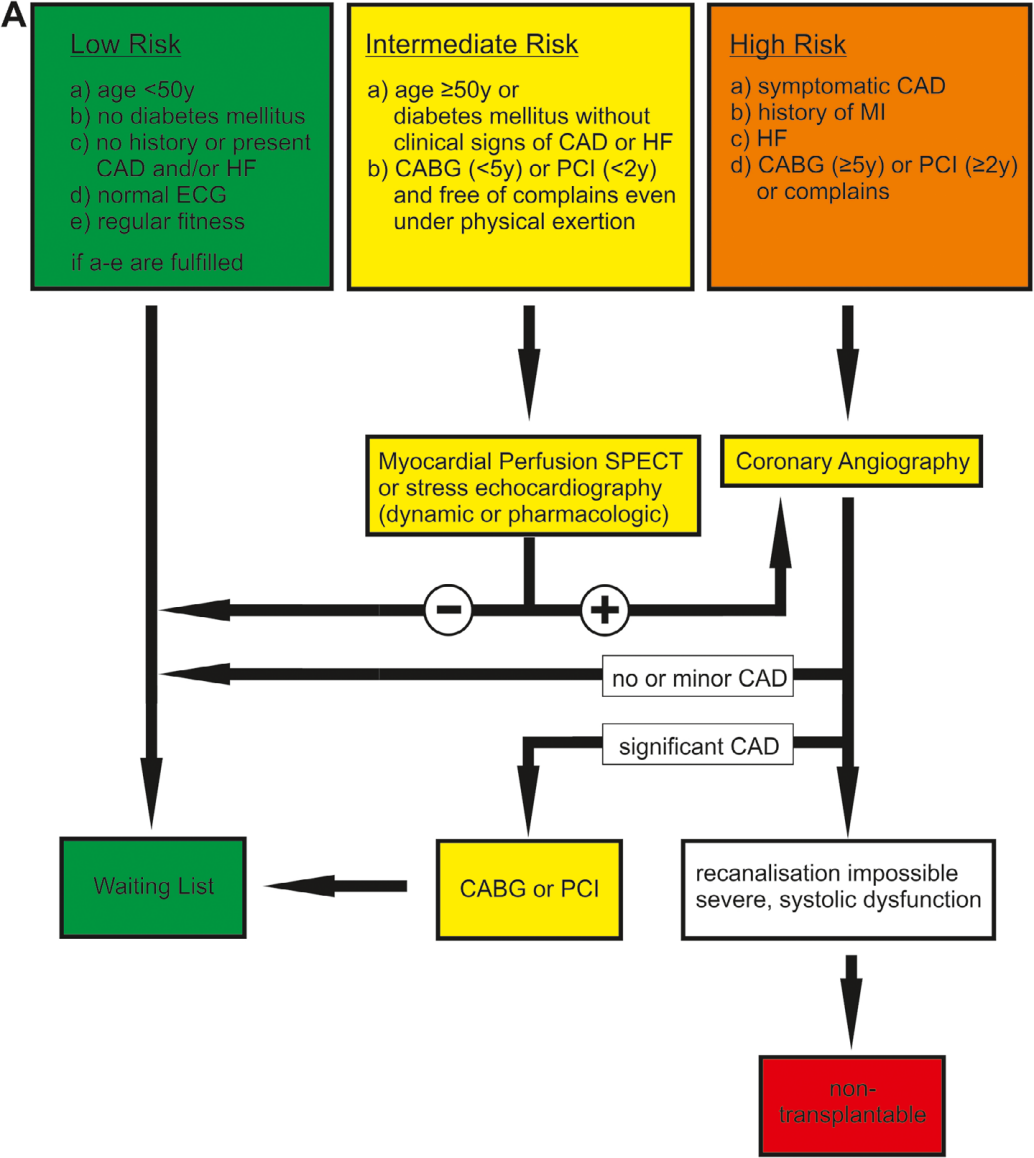
New MCRSS. Figure was adapted from [15]. Patients are categorized into three cardiovascular risk groups: low, intermediate, and high. CABG, coronary artery bypass graft; CAD, coronary artery disease; ECG, electrocardiogram; HF, heart failure; MCRSS, Münster Cardiovascular Risk Stratification Score; MI, myocardial infarction; PCI, percutaneous coronary intervention; SPECT, single photon emission computed tomography; y, years.

Figure 1 shows EFS after listing. Figure 2 depicts EFS according to the risk groups classified by MCRSS. Interestingly, neither imaging results from DSE nor from MPS predicted EFS if patients from all MCRSS risk groups (with and without CAD) were included in the analysis. However, analyzing MCRSS subgroups (Figure 3) led to interesting observations. Most strikingly, perfusion deficits in MPS (SSS >0%–10%) were associated with an HR of 3.14 ([CI 1.35–7.29], *p* = 0.0078) in the MCRSS low‐risk group (Figure 3A), whereas no significant incremental prognostic value of MPS was found in the MCRSS intermediate risk group, the HR for SSS 1 > 0%–10% was 1.15 ([CI 0.58–2.3], *p* = 0.6936) and 1.74 ([CI 0.70–4.32], *p* = 0.2300) for SSS >10%; (Figure 3B). In the MCRSS high‐risk group (Figure 3C), the HR for SSS 1%–10% was 1.03 ([CI 0.30–3.58], *p* = 0.9599), and 1.43 ([CI 0.55–3.69], *p* = 0.4650) for SSS >10%, however, with no statistical significance. The MCRSS subgroup analysis for DSE showed an HR of 1.27 ([CI 0.30–5.45], *p* = 0.7480) for low‐risk patients with stress‐depended wall motion abnormalities detected by DSE (Figure 3D), HR 1.76 ([CI 0.62–4.9], *p* = 0.2869) for intermediate‐risk patients (Figure 4E) and HR 2.39 ([CI 0.76–7.5], *p* = 0.1351) for high‐risk patients (Figure 4F), all not showing statistical significance.

## 4 Discussion

There is consensus that the optimal CV status of recipients should be sought before transplantation, which requires CV assessment in candidates with the major limitation that such CV assessment is likely to be performed years before KTx. Furthermore, guideline recommendations regarding CV assessment in kidney transplant candidates are limited by the lack of prospective data and therefore vary [13, 22–24]. It has often been criticized that noninvasive tests for CAD in patients with ESRD have imperfect sensitivity and specificity [8]. However, a recent meta‐analysis suggested that the prognostic value of an abnormal DSE or MPS is as valuable as an abnormal coronary angiography for predicting CV mortality and major adverse cardiac events (MACEs) in these patients [25].

Different CV diagnostic algorithms are followed. More aggressive strategies, including invasive diagnostic testing for all patients, are contrasted with noninvasive test‐based or exam‐based approaches, depending on the patient's symptomatic status [13]. This is important because CV EFS is not different between wait‐list patients with normal coronary arteries, nonobstructive CAD, or obstructive CAD with intervention, but is significantly better than that of patients with obstructive CAD without intervention [9, 26]. This benefit has not been consistently demonstrated, and many authors do not recommend CAD‐based screening in waiting list candidates [2, 27–29].

In general, a risk‐based stratification of patients seems reasonable to maintain the balance between missing a diagnosis and excessive diagnostics, both putting the patients at risk. This strategy is supported by recent guidelines [13, 22]. Kasiske et al. stated that risk‐stratified screening effectively avoids unnecessary testing in >40% of waiting list candidates [30]. Thus, one can follow the idea of simplified screening–equations‐based strategies–to obtain a CVD‐risk assessment that should lead to individualized stratification of transplant candidates allowing for efficient and cost‐effective evaluation. This was pursued in a previous study where we showed that the CV risk assessment score MCRSS predicted event‐free and overall survival in our waiting list candidates [7]. Between 2006 and 2009, we mainly followed the recommendations of the MCRSS regarding the CV evaluation of transplant candidates. Consequently, only a minority of patients had been tested by DSE or MPS (28/347, 12.4%) [7].

It is generally assumed that particularly young patients on the KTx waiting list belong to a group of low‐risk candidates and do not require intensive CV workup, while those with angina pectoris or history of CV events should undergo coronary angiography [6]. Interestingly, the age of low‐risk patients with normal MPS was not different from those with pathological findings (38 ± 9.1 vs. 41 ± 7.9 years). On the other hand, it is also widely accepted to screen all older, even asymptomatic patients with either diabetes or other risk factor for CAD by MPS or DSE [6, 22]. Especially in diabetic transplant candidates, the prognostic value of DSE and MPS was shown, but the value of these modalities has been reported with a wide range of sensitivities and specificities [6, 31, 32]. According to the literature, sensitivity and specificity for detecting CAD in renal patients vary between 0.29 and 0.92 (sensitivity) and 0.67 and 0.89 (specificity) for MPS; sensitivity of DSE ranges between 0.44 and 0.89 and specificity between 0.71 and 0.94 [32]. The reported agreement rate of both techniques is between 68% and 79% [33]. Poor image quality can be problematic herein, occurring in up to 30% of patients due to excessive heart movement caused by hyperventilation and tachycardia, and in up to 10% due to patient‐related factors such as obesity and lung disease [33]. However, the use of ultrasound contrast agents may increase the rate of adequate visualization. Discrepancies between methods may also occur when hypokinetic or akinetic segments are considered. This difference is considered to be a consequence of the different mechanisms of viability detection by the two assessment techniques. For example, a higher degree of functional integrity of myocytes is naturally required for contractile reserve than for radiotracer uptake by cells [34].

Here, we aimed to improve the diagnostic workup (identification of the most reasonable approach) and ultimately the survival of our patients on the KTx waiting list by adding noninvasive technical examinations, namely DSE and MPS. At the time of evaluation, 11.2% of patients had a known history of CAD.

Noninvasive stress testing was performed in 350 patients; stress‐induced ischemia was present in 11.1% of MPS and 10.7% of DSE investigations leading to interventions in 30% of cases (approx. 6.2% of all cases). This is in contrast to the observations of Gowdak et al. who found significant stenosis (>70% luminal narrowing) in 45.2% of unselected waiting list candidates in Brazil [2]. However, this rate seems to be unusually high, as other studies report noticeably lower prevalence comparable to our results [28, 29]. Moreover, our results are consistent with data showing that only 2.9%–9.5% of patients with pretransplant stress testing proceed to coronary intervention [13]. Overall survival was good (71.9%) and comparable to our previous analysis with a lower CV event rate (2.43 CV events vs. 5.89 CV events/100 person‐years) [7]. In this cohort, the MCRSS classification in general predicted EFS (Figure 2).

From these data, it can be concluded that the systematic use of DSE and MPS followed by coronary angiography in cases of pathological findings on previous stress tests does not necessarily identify the transplant candidate with the highest probability of developing a CV event (in contrast to MCRSS). In addition to classic CAD‐related CV events, CV events in patients with ESRD are often related to microvascular complications, which are likely to be missed by these conventional techniques focusing on macrovascular characteristics [3]. In line with this, Helve et al. observed that revascularization was not associated with improved survival in their transplant candidates [14]. Additionally, the recent ISCHEMIA‐CKD study showed no superiority of an initial invasive strategy to reduce mortality or myocardial infarction in patients with stable CAD and advanced CKD [10]. However, according to Helve et al. and Wang et al., we found that reversible defects in MPS as well as both fixed and reversible abnormalities predicted the CV outcome, especially in low‐risk patients [14, 25]. Moreover, our findings are consistent with those of a recent meta‐analysis showing that abnormal myocardial perfusion on stress MPS is strongly associated with adverse cardiovascular events in CKD patients [35]. These results underscore the importance of including MPS in risk stratification protocols for ESRD patients. Contrarily, DSE was not predictive in the same cohort. Thus, we conclude that MPS should be added to the MCRSS classification system, especially in the low‐risk group to improve individualized risk stratification (Figure 4). The exact treatment strategy, including the best retesting and its frequency, remains the subject of future investigations. Furthermore, the value of MPS in a larger MCRSS high‐risk group should be tested in further studies, as we show here a clear trend toward a prognostic impact of MPS in this cohort.

New approaches addressing the functionality of the microvasculature, for example, by quantitative assessment of the coronary flow reserve (e.g., PET), may be promising here [36, 37].

### 4.1 Study Limitations

Although this is one of the largest prospective studies comparing MPS and DSE in kidney transplant candidates for CV risk assessment with long‐term follow‐up, it is a nonrandomized, single‐center, observational study with all the inherent limitations such as the presence of confounders of such a study design. However, this study design should not influence the results of invasive or noninvasive assessments. Although the study protocol included MPS and DSE tests for each patient, individual tests were missing for some patients; therefore, complete data are not available for all, but for most patients. Although our findings indicate limited prognostic value for DSE compared to MPS, this may be influenced by the operator dependency and challenges in achieving target heart rates, particularly in diabetic patients. Literature suggests that when quality criteria are met, DSE achieves high predictive accuracy. Future studies should aim to standardize quality metrics and evaluate their impact on predictive performance in ESRD patients. Another limitation is the lack of additional information on the hemodynamic significance of the coronary lesion (e.g., by fractional flow reserve) and quantitative characteristics of the coronary wall and lumen (e.g., by intravascular ultrasound). Moreover, results should be considered exploratory from a statistical standpoint, and subgroup analyses may not be able to detect all potential associations between risk variables due to reduced power.

We included patients at the time of listing into the study. Although this reflects the real‐world approach, patients vary widely in age and time from the onset of CKD and dialysis to the time of evaluation. It has been speculated that CKD affects the pathology and presentation of CAD in a time‐ and age‐dependent manner [8]. Despite different patient histories, we found an incremental prognostic value of MPS using SSS categories. A potential limitation of MPS regarding microvascular function is the lack of absolute quantification of perfusion reserve. Since DSE is highly dependent on the experience of the examiner, this could be a methodological limitation, although two highly experienced physicians performed all investigations.

## 5 Conclusion

We conclude that stress testing is sufficient as a first diagnostic test for patients with a low pretest probability of CAD based on MCRSS. In patients with low MCRSS risk scores, MPS, but not DSE, provides incremental prognostic value compared with the current MCRSS risk score. Based on our study data, we have developed a better risk estimator, the modified MCRSS, which is now routinely used for MPS in ESRD patients with low CV risk.

## Conflicts of Interest

The authors declare no conflicts of interest.

## Supporting information



Supporting Information

## Data Availability

The data that support the findings of this study are available from the corresponding author upon reasonable request. The data are not publicly available due to privacy or ethical restrictions.
