# Supplementary material for: Myocardial Perfusion Scintigraphy Provides Incremental Prognostic Value in Patients on the Kidney Transplant Waiting List
Source: Clin Transplant. 2025 Feb 21;39(2):e70114. doi: 10.1111/ctr.70114 (PMC11843186; doi:10.1111/ctr.70114)
Supplement: Supplementary file 1 — Supporting Information [file CTR-39-e70114-s001.docx]

Here we present five different patients who are categorized into their corresponding MCRSS risk category using the flowchart in Figure 4.

1. Patient: 42-year-old woman with normal electrocardiogram findings, regular fitness, no diabetes mellitus and no history of coronary artery disease and heart failure.
2. Patient: 63-year-old woman with normal electrocardiogram findings, regular fitness, no diabetes mellitus and no history of coronary artery disease and heart failure.
3. Patient: 69-year-old man with a history of myocardial infarction and coronary artery bypass graft 4 years ago
4. Patient: 69-year-old man with a history of myocardial infarction and percutaneous coronary intervention 1 years ago
5. Patient: 69-year-old man who underwent coronary artery bypass graft 4 years ago without a history of myocardial infarction. Free of complains even under physical exertion

Supplemental Table 1.

**S1 Table. MCRSS patient examples.**

| **MCRSS risk** | low | intermediate | high |
| --- | --- | --- | --- |
|  | a) age <50y  b) no diabetes mellitus  c) no history or present  CAD and/or HF  d) normal ECG  e) regular fitness | a) age ≥50y or diabetes  mellitus without  clinical signs of CAD  or HF  b) CABG (<5y) or   PCI (<2y) and free  of complains even  under physical  exertion | a) symptomatic CAD  b) history of MI  c) HF  d) CABG (≥5y) or   PCI (≥2y) or   complains |
| Patient 1 | X |  |  |
| Patient 2 |  | X |  |
| Patient 3 |  |  | X |
| Patient 4 |  |  | X |
| Patient 5 |  | X |  |

y: years, CAD: coronary artery disease, HF: heart failure, ECG: electrocardiogram, MI: myocardial infarction,
PCI: percutaneous coronary intervention, CABG: coronary artery bypass graft

**
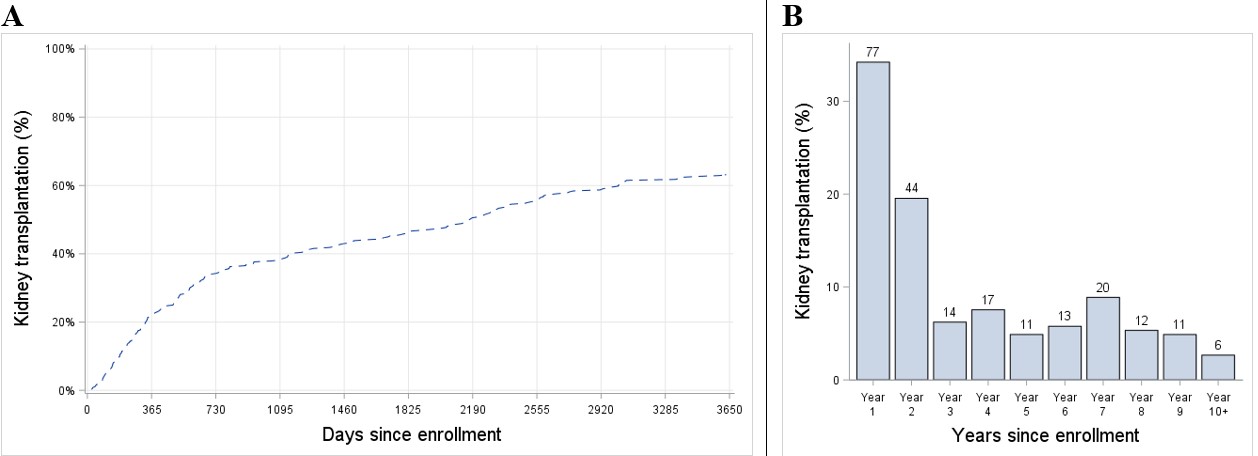
**

**S1 Fig. Time from listing to KTx.** Percent of patients transplanted after listing for transplantation (A). Absolute numbers are given in (B) above the columns.
